# DHA Protects Hepatocytes from Oxidative Injury through GPR120/ERK-Mediated Mitophagy

**DOI:** 10.3390/ijms22115675

**Published:** 2021-05-26

**Authors:** Jinglong Chen, Danping Wang, Yibo Zong, Xiaojing Yang

**Affiliations:** MOE Joint Key Laboratory of Animal Physiology and Biochemistry, Nanjing Agricultural University, Nanjing 210095, China; 2012207005@stu.njau.edu.cn (J.C.); 2018107024@stu.njau.edu.cn (D.W.); 2019207008@stu.njau.edu.cn (Y.Z.)

**Keywords:** oxidative stress, liver injury, DHA, mitophagy, ERK1/2 signaling

## Abstract

Oxidative stress occurs in a variety of clinical liver diseases and causes cellular damage and mitochondrial dysfunction. The clearance of damaged mitochondria by mitophagy may facilitate mitochondrial biogenesis and enhance cell survival. Although the supplementation of docosahexaenoic acid (DHA) has been recognized to relieve the symptoms of various liver diseases, the antioxidant effect of DHA in liver disease is still unclear. The purpose of our research was to investigate the antioxidant effect of DHA in the liver and the possible role of mitophagy in this. In vitro, H_2_O_2_-induced injury was caused in AML12 cells. The results showed that DHA repressed the level of reactive oxygen species (ROS) induced by H_2_O_2_ and stimulated the cellular antioxidation response. Most notably, DHA restored oxidative stress-impaired autophagic flux and promoted protective autophagy. In addition, PINK/Parkin-mediated mitophagy was activated by DHA in AML12 cells and alleviated mitochondrial dysfunction. The ERK1/2 signaling pathway was inhibited during oxidative stress but reactivated by DHA treatment. It was proven that the expression of ERK1/2 was involved in the regulation of mitophagy by the ERK1/2 inhibitor. We further proved these results in vivo. DHA effectively alleviated the liver oxidative damage caused by CCl_4_ and enhanced antioxidation capacity; intriguingly, autophagy was also activated. In summary, our data demonstrated that DHA protected hepatocytes from oxidative damage through GPR120/ERK-mediated mitophagy.

## 1. Introduction

A large number of studies have demonstrated that most clinical liver disease is associated with an increasing oxidative stress injury, including NAFLD (non-alcoholic fatty liver disease) and NASH (non-alcoholic steatohepatitis) [1,2]. Oxidative stress is based primarily on the destruction of intracellular redox states. Subsequent excessive production of reactive oxygen species (ROS) would damage cellular components and then induce hepatocyte apoptosis [3]. ROS are highly reactive radicals that are normally generated in the mitochondria; they impair mitochondrial function during redox imbalance conditions. Previous studies also found that mitochondrial dysfunction and the activation of hepatic stellate cells caused by ROS accelerate the progression of NAFLD to fibrosis or HCC (hepatocellular carcinoma) [4,5]. Thus, maintaining the balance of redox status and eliminating damaged mitochondria are effective methods for alleviating oxidative damage to hepatocytes and reducing the progression of liver disease.

(DHA), an ω-3 long-chain polyunsaturated fatty acid, is an important fatty acid that is present in fish oil [6]. Some studies have shown that DHA could effectively protect against neurodegenerative diseases, such as Alzheimer’s disease, and cardiovascular diseases through activating autophagy to regulate oxidative injury [7,8]. In the liver, supplementation with DHA has been shown to improve the symptoms of various liver diseases [9,10]. Recent studies have indicated that supplementation with DHA effectively relieves NAFLD and NASH by activating the function of the GPR120 (a long-chain fatty acid receptor, FFAR4)-mediated anti-inflammation pathway and inhibiting lipid accumulation in hepatocytes [11,12]. However, it remains unclear whether ω-3 PUFA are effective at counteracting oxidative stress in the liver [13]. The mechanism by which oxidative stress can be reduced following ω-3 PUFA supplementation needs to be better understood.

Mitophagy is an autophagic pathway that regulates mitochondrial number, and performs quality control to remove damaged mitochondria [14,15]. Regulating the process of mitophagy is important for alleviating oxidative damage to hepatocytes and reducing the progression of liver disease [16]. Mitophagy utilizes the same core autophagy machinery as other forms of selective autophagy. The most widely studied signaling-pathway-regulating mitophagy is PINK/Parkin-dependent [17,18]. Under external stimulation, PINK1 would be located on outer mitochondrial membrane (OMM) and promotes the recruitment of Parkin E3 ubiquitin ligase [19]. Parkin ubiquitinates several outer membrane components. Thereafter, the phosphorylated polyubiquitin chains on mitochondrial are recognized by p62/SQSTM1 and activate autophagosome formation by binding to LC3 [20]. Another study demonstrated that the expression of Parkin is down-regulated in the liver during NAFLD; on the contrary, reversing Parkin-related mitophagy could protect mitochondria and hepatocytes from HFD-induced oxidative injury [21]. Whether DHA could promote mitophagy and alleviate oxidative liver damage, and the underlying molecular mechanism remains largely undetermined.

Therefore, in the present study, we analyzed the effects of DHA on hepatocytes, especially in terms of protecting them from oxidative damage, and investigated the possible mechanisms. These results showed that DHA can relieve oxidative damage in the liver by activating GPR120/ERK cascade signaling pathway-mediated mitophagy. The results increase our understanding of DHA antioxidant effects in the liver and may aid in the development of new strategies for the treatment of liver diseases.

## 2. Results

### 2.1. DHA Activated the Cellular Antioxidant Response and Alleviated the Oxidative Damage Caused by H_2_O_2_ in Hepatocytes

**Figure 1:**
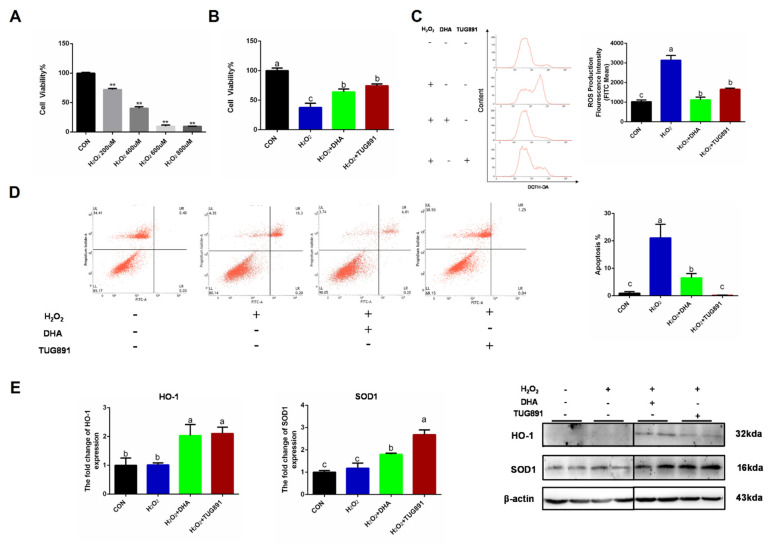
Docosahexaenoic acid (DHA) activated the cellular antioxidant response and alleviated the oxidative damage caused by H_2_O_2_ in hepatocytes. AML12 cells were pre-treated with DHA (50 μM) and TUG891 (10 μM) for 12 h, and then treated with H_2_O_2_ (400 μM) for another 2 h. (**A**,**B**) The cell viability of AML12 cells after treated with H_2_O_2_ and DHA was detected by CCK-8 assay kit. (**C**) Cellular reactive oxygen species (ROS) was stained with DCFH-DA fluorescent probe and detected by flow cytometry. (**D**) The apoptosis of AML12 cells was evaluated with Annexin V-FITC Apoptosis Staining kit. (**E**) The protein expression of Superoxide dismutase1 (SOD1) and Heme Oxygenase-1 (HO-1) was determined by Western-blotting. All data are expressed as mean ± SEM of three independent experiments. Significant differences between each group are shown with different letters (e.g., a, b, and c mean a statistically significant difference between each other, ** *p* < 0.05).

Oxidative stress was induced in AML12 cells using H_2_O_2_ (400 uL/2 h), and pretreating the cells with DHA or a GPR120 agonist for 12 h. We found that the decreased cell viability and apoptosis could be effectively alleviated by DHA treatment (Figure 1A,B,D). H_2_O_2_ damaged AML12 cells mainly by producing excessive ROS-damaging organelles. Therefore, we tested the ROS content and found that DHA and TUG891 (a potent and selective agonist for GPR120) effectively inhibited the production of ROS (Figure 1C). Antioxidant-related protein expression of SOD1 (superoxide dismutase) and HO-1 (heme oxygenase-1), as the major enzymes for ROS clearance in vivo, were also significantly increased (Figure 1E). These results suggest that DHA and TUG891 enhance the scavenging ability of ROS in hepatocytes with oxidative damage and increase the ability to counteract stress.

### 2.2. The Effect of DHA on Scavenging ROS Is Associated with the Restoration of Protective Autophagy

**Figure 2:**
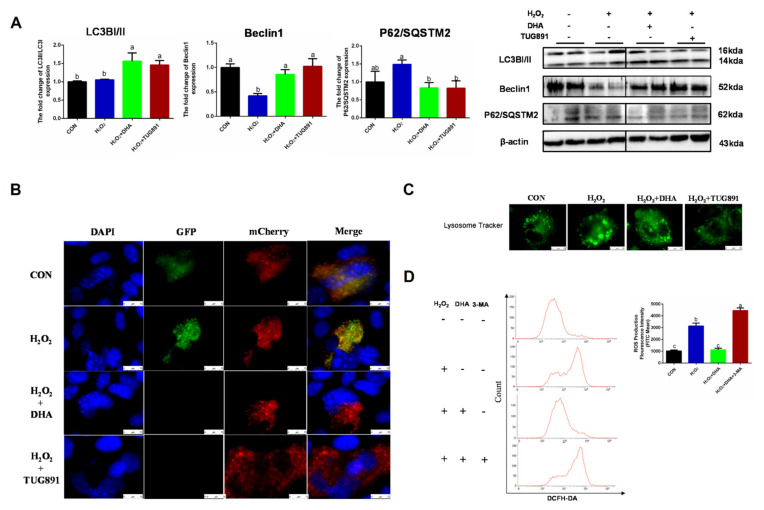
The effect of DHA on scavenging ROS is associated with restoration of protective autophagy. AML12 cells were pre-treated with DHA (50 μM) and TUG891 (10 μM) for 12 h, and then treated with H_2_O_2_ (400 μM) for another 2 h. (**A**) The protein expression of LC3II, Beclin1, and P62/SQSTM2 was determined by Western blotting. (**B**) The autophagic flux was detected by using confocal microscopy after transfection of GFP-mCherry-LC3II. (**C**) The lysosomes in AML12 cells were stained with LysoSensor™ and detected by using confocal microscopy after treatment. (**D**) Cells were pre-treated with 3-Methyladenine (3-MA) (5 mM) for 2 h, and then treated with DHA (50 μM) for 12 h followed by H_2_O_2_ (400 μM) 2 h challenge. Cellular ROS was stained with DCFH-DA fluorescent probe and detected by flow cytometry. All data are expressed as mean ± SEM of three independent experiments. Significant differences between each group are shown with different letters (e.g., a, b, and c mean a statistically significant differences between each other, *p* < 0.05).

Autophagy is a critical intracellular self-protective mechanism that promotes scavenging and renewal in damaged organelles. The expression of LC3II is necessary for autophagosome formation and is used as a major marker of autophagy. Beclin1 is involved in the initiation and regulation of autophagy, while P62 delivers ubiquitin-binding protein complexes to autophagosomes and promotes degradation through an autophagosome lysosome fusion mechanism. In our study, DHA and TUG891 promoted Beclin1 expression and LC3II transformation, while the degradation of P62 increased, suggesting autophagy (Figure 2A). Since LC3II can accumulate as a result of autophagy initiation or impaired autophagy flux, we evaluated the autophagy flux by transfecting the mChreey-GFP-LC3 plasmid, and the results also showed that H_2_O_2_ blocked the autophagy flux, which was restored by DHA pretreatment (Figure 2B). We also observed that the lysosome volume increased and the number of lysosomes decreased in the H_2_O_2_ group (Figure 2C). This suggested that the lysosomal function was impaired by oxidative stress. In order to further verify the role of autophagy in DHA-induced antioxidative damage, we inhibited the autophagy with autophagy inhibitor 3-MA (3-methyladenine). As expected, when autophagy was blocked, DHA could not effectively alleviate ROS production (Figure 2D). Therefore, we conclude that DHA and TUG891 can alleviate the oxidative damage of liver cells by activating autophagy.

### 2.3. DHA Reversed H_2_O_2_-Induced Mitochondrial Injury by Promoting PINK/Parkin-Mediated Mitophagy in Hepatocytes

**Figure 3:**
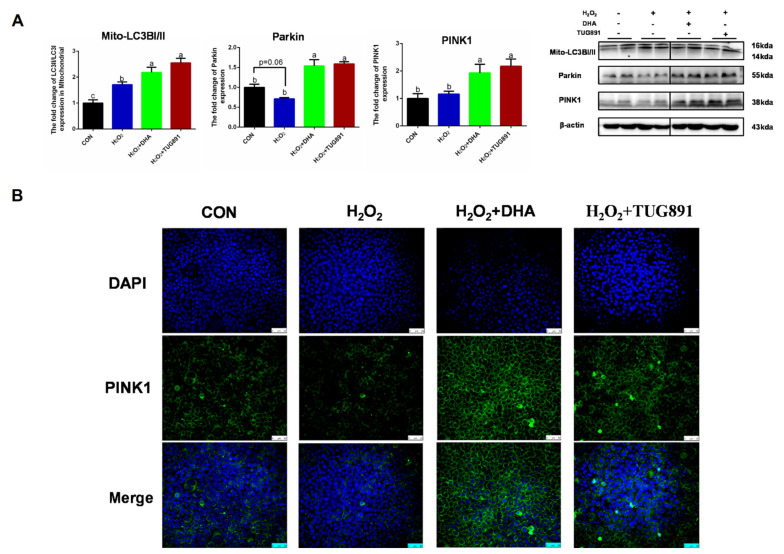
DHA against H_2_O_2_-induced hepatocyte injury though PINK/Parkin-mediated mitophagy. AML12 cells were pre-treated with DHA (50 μM) and TUG891 (10 μM) for 12 h, and then treated with H_2_O_2_ (400 μM) for another 2 h. (**A**) The protein expression of PINK1, Parkin, and Mito-LC3II was determined by Western-blotting. (**B**) The immunofluorescence detected the expression of PINK in AML12 cells. All data are expressed as mean ± SEM of three independent experiments. Significant differences between each group are shown with different letters (e.g., a, b, and c mean a statistically significant differences between each other, *p* < 0.05).

Mitophagy is considered an important mechanism for clearing damaged mitochondria and maintaining mitochondrial homeostasis. To evaluate PINK/Parkin-mediated mitophagy, we detected the expression of PINK, Parkin, and Mito-LC3. A WB assay showed significant increases in PINK1, Parkin, and Mito-LC3 expression in AML12 cells following DHA treatment (Figure 3A). To confirm these results, the intercellular expression of PINK was visualized by immunofluorescence (Figure 3B). These data demonstrate that PINK1/Parkin-mediated mitophagy was activated by DHA to protect AML12 cells from oxidative injury.

**Figure 4:**
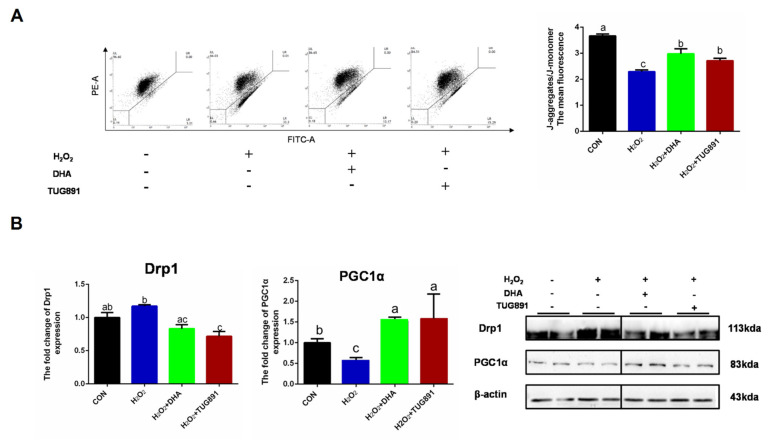
DHA reversed H_2_O_2_-induced mitochondrial injury in hepatocytes. AML12 cells were pre-treated with DHA (50 μM) and TUG891 (10 μM) for 12 h, and then treated with H_2_O_2_ (400 μM) for another 2 h. (**A**) The mitochondrial membrane potential (ΔΨm) was detected with JC-1 staining assay. (**B**) The protein expression of Drp1 and PGC1α was determined by Western-blotting. All data are expressed as mean ± SEM of three independent experiments. Significant differences between each group are shown with different letters (e.g., a, b, and c mean a statistically significant differences between each other, *p* < 0.05).

To investigate the mitochondrial function during oxidative damage, integrity of mitochondria was monitored by JC-1 probe detecting. In normal mitochondria with high ΔΨm, JC-1 forms complexes known as J-aggregates. While in damaged mitochondria with low ΔΨm, JC-1 remains in the monomeric form. When excited at 510 nm, JC-1 monomers emit a green fluorescence with a maximum at 527 nm. Aggregates of JC-1 emit an orange-red fluorescence with a maximum at 590 nm. The result showed a significant reduction in the function of mitochondria in AML12 cells with H_2_O_2_ treatment, while DHA had a significant mitigating effect on mitochondrial depolarization during oxidative stress (Figure 4A). Mitochondrial-dynamics-related protein Drp1 and biosynthesis-related protein PGC1α were also detected by Western blotting. The results showed that DHA promoted mitochondrial synthesis and inhibited mitochondrial fission (Figure 4B). This indicated that DHA was involved in mitochondrial quality control and maintaining mitochondrial function.

### 2.4. Inhibiting ERK1/2 Signaling Decreased the Effect of DHA against Oxidative Stress

**Figure 5:**
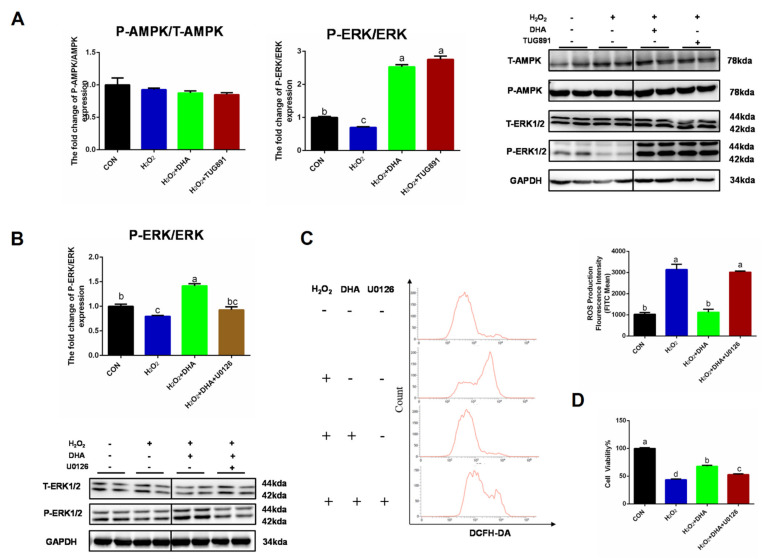
Inhibiting ERK1/2 signaling decreased the effect of DHA against oxidative stress. AML12 cells were pre-treated with DHA (50 μM) and TUG891 (10 μM) for 12 h, and then treated with H_2_O_2_ (400 μM) for another 2 h. (**A**) The protein expression of ERK1/2, P-ERK1/2, AMPK, and P-AMPK was determined by Western-blotting. (**B**) The protein expression of ERK1/2 and P-ERK1/2 in AML12 cells after treated with U0126. (**C**) Cells were pre-treated with U0126 (10 μM) for 2 h, and then treated with DHA (50 μM) for 12h followed with H_2_O_2_ (400 μM) 2h challenge. Cellular ROS was stained with DCFH-DA fluorescent probe and detected by Flow cytometry. (**D**) The cell viability of AML12 cells. All data were expressed as mean ± SEM of three independent experiments. Significant differences between each group are shown with different letters (e.g., a, b, and c mean a statistically significant differences between each other, *p* < 0.05).

We further investigated the specific mechanism of DHA’s regulation of autophagy. AMPK and ERK1/2 are recognized as major signaling pathways involved in autophagy regulation. Western blotting results showed no significant change in P-AMPK/AMPK expression. However, the phosphorylation level of ERK1/2 was significantly inhibited by H_2_O_2_, and pre-treatment with DHA could significantly increase the phosphorylation level of ERK1/2 (Figure 5A). We hypothesized that DHA might promote an increase in autophagy by activating the ERK1/2 signaling pathway. Therefore, we inhibited the expression of ERK1/2 with the specific inhibitor U0126 (HY-12031A, MCE, Shanghai, China). The results showed that with the inhibition of ERK1/2 expression (Figure 5B), DHA could not effectively improve H_2_O_2_-induced hepatocyte injury or ROS production (Figure 5C,D).

### 2.5. Inhibition of ERK1/2 Signaling Blocked DHA-Mediated Mitophagy

**Figure 6:**
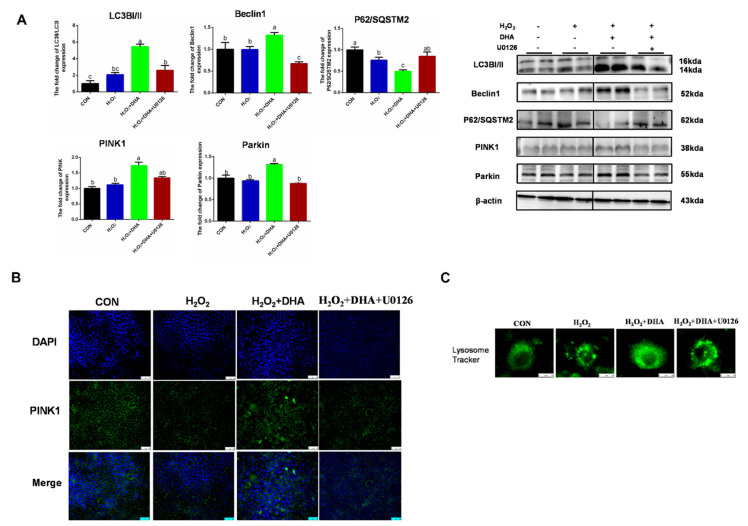
The inhibition of ERK1/2 signaling blocked DHA-mediated mitophagy. Cells were pre-treated with U0126 (10 μM) for 2 h, and then treated with DHA (50 μM) for 12 h followed with H_2_O_2_ (400 μM) 2h challenge. (**A**) The protein expression of Beclin1, P62/SOSTM1, LC3B, PINK, and Parkin was determined by Western-blotting. (**B**) The immunofluorescence detected the expression of PINK in AML12 cells. (**C**) The lysosomes in AML12 cells were stained with LysoSensor™ and detected by using confocal microscopy after treatment. All data are expressed as mean ± SEM of three independent experiments. Significant differences between each group are shown with different letters (e.g., a, b, and c mean a statistically significant differences between each other, *p* < 0.05).

To further investigate the role of ERK1/2 in DHA-induced protective mitophagy, U0126 was utilized to repress the phosphorylation of ERK1/2. The inhibition of ERK1/2 decreased LC3 and Beclin1 expression and blocked the degradation of P62/SQSTM1, which showed that the formation or degradation of autophagosomes induced by DHA required the participation of ERK1/2 (Figure 6A). Then we evaluated the impact of ERK1/2 inhibition on mitophagy. The blocking of ERK1/2 signaling abolished the DHA-induced enhancement of PINK or Parkin expression (Figure 6A,B), and decreased the protective effect of DHA on lysosomal function (Figure 6C). Therefore, the above results demonstrate that the ERK1/2 signaling pathway may be indispensable for DHA-regulated mitophagy and anti-oxidative stress function.

### 2.6. Supplementation with DHA against CCl_4_-Induced Liver Injury in Mice by Activating Autophagy

**Figure 7:**
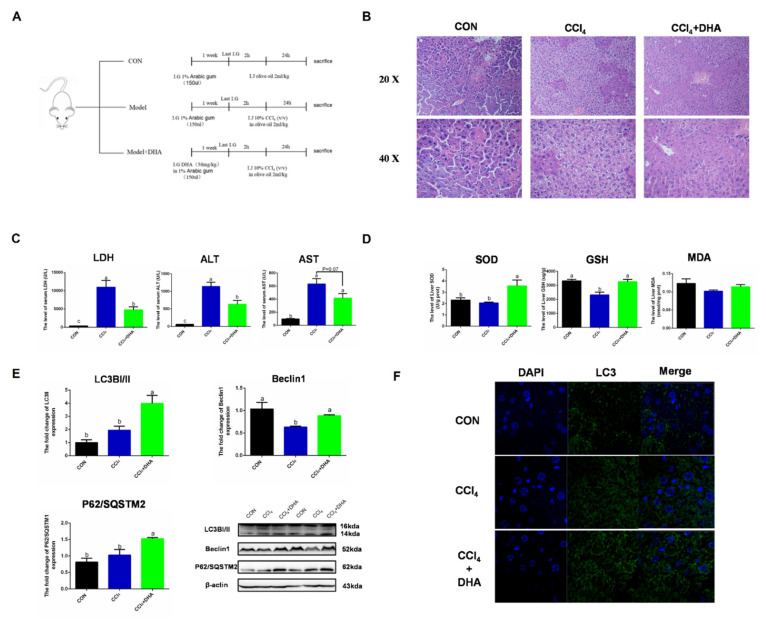
Supplements of DHA protect against CCl_4_-induced liver injury in mice by activating autophagy. (**A**) Experimental design All mice were randomly divided into three groups (*n* = 10). (**B**) The HE stains images of liver. (**C**) The level of lactate dehydrogenase (LDH), aspartate aminotransferase (AST), and alanine aminotransferase (ALT) in serum. (**D**) The activities of SOD and glutathione (GSH) in liver. (**E**) The protein expression of Beclin1, P62/SOSTM1, and LC3B in liver were determined by Western-blotting. (**F**) The immunofluorescence detected the expression of LC3B in liver. The data are expressed as mean ± SEM and the significant differences between each group are shown with different letters (e.g., a, b, and c mean a statistically significant differences between each other, *p* < 0.05).

To verify the effect of DHA on the liver, CCl_4_-induced acute liver injury was utilized as an oxidative injury model in vivo. Due to CCl_4_ being metabolized by CYP450 to form free radicals (CCl_3_^−^ and CCl_3_O^2−^) in the liver, triggering intercellular oxidative damage, this model has been widely used in the study of the mechanisms of acute liver injury. In order to assess the protective effect of DHA on the liver, we used mice with intraperitoneal injection of CCl_4_ as a liver injury model [22]. HE staining results showed that the normal morphology of hepatocytes was destroyed in the CCl_4_ group, while the liver injury was relieved by DHA treatment (Figure 7B). In the model group, the levels of AST (aspartate transaminase), ALT (alanine transaminase), and LDH (lactate dehydrogenase) were significantly increased and the DHA-treated group showed effective mitigation (Figure 7C). DHA also increased the activity of SOD and GSH in the liver (Figure 7D). As expected, the expression of LC3II and Beclin1 significantly increased due to DHA and this was further confirmed by immunofluorescence (Figure 7E,F). However, the protein expression of P62/SQSTM1 increased in the DHA group. Similar results were also reported in another study, which may be because DHA can promote the transcription and degradation of P62. When the transcription rate is greater than the degradation level, the protein expression of P62 will increase [23]. Above all, we confirmed, in vivo, that DHA can effectively alleviate the liver oxidative damage caused by CCl_4_, which may be related to the activation of autophagy.

## 3. Discussion

**Figure 8:**
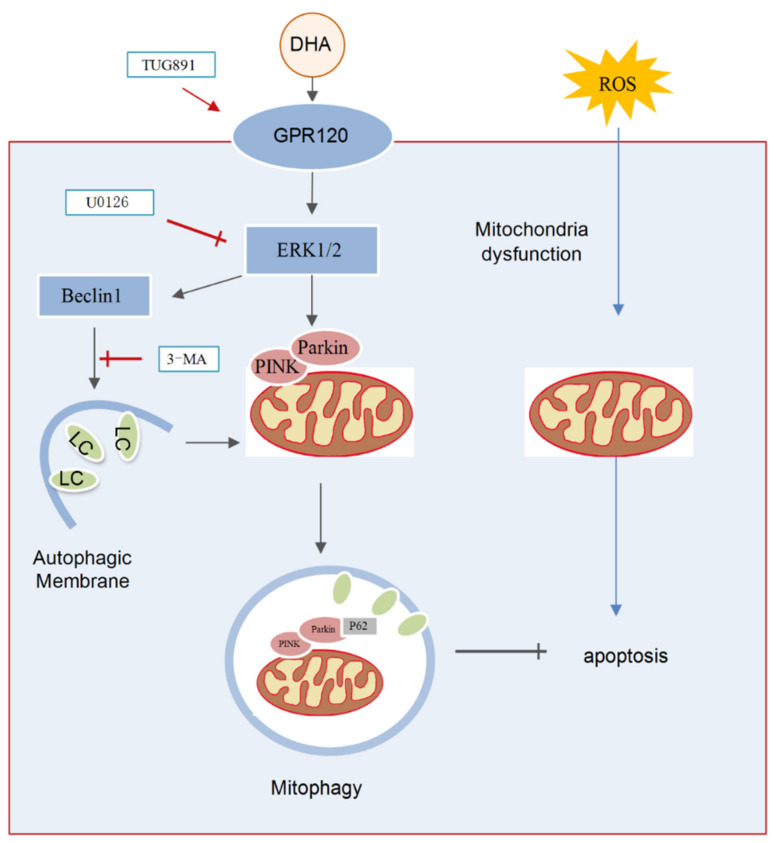
The schematic representation shows that DHA attenuates H_2_O_2_-induced hepatocyte injury through ERK1/2-dependent mitophagy. Oxidative stress would induce hepatocyte apoptosis by impairing mitochondrial function. DHA and TUG891 (a agonist of GPR120) supplementation activates GPR120/ERK1/2 signaling pathway which regulates PINK1/Parkin-mediated mitophagy. The up-regulation of protective mitophagy level promotes mitochondrial renewal and maintains mitochondrial homeostasis during oxidative injury. However, inhibiting the activation of ERK1/2 and autophagy by selective inhibitor U0126 or 3-MA, respectively, could block the protective effect of DHA.

In the present study, we demonstrated that DHA supplementation can effectively attenuate the oxidative stress caused by liver injury. The specific mechanism included the following: (1) DHA evoked an antioxidation response in hepatocytes and scavenged the excess ROS produced by H_2_O_2_ and (2) oxidative stress significantly decreased the level of MMP and induced mitochondria dysfunction, while, DHA activated mitophagy through the GPR120/ERK pathway, thereby improving mitochondrial quality and alleviating liver damage and demonstrating that DHA/GPR120-activated ERK1/2 signaling may be involved in PINK/Parkin-mediated mitophagy (Figure 8).

As a crucial organ, the liver is easily harmed by oxidative stress. Many studies have indicated that DHA up-regulates the expression of HO-1 or SOD to relieve oxidative stress in neuronal cells, and is involved in the treatment of a variety of neurodegenerative diseases [24]. However, its antioxidant function in hepatocytes has been less reported. Our data showed that DHA and TUG891 (an agonist of GPR120) treatment effectively alleviated H_2_O_2_-induced oxidative damage, decreasing the ROS level and apoptosis in the AML12 cell line. Moreover, DHA up-regulated the expression of downstream protein SOD1 and HO-1. This suggests that H_2_O_2_ triggers oxidative injury, and DHA protection hepatocytes may be associated with antioxidant response.

Autophagy is actively involved in the cellular antioxidative response and has a complex interaction with oxidative stress. Some researchers have reported that the excessive ROS produced during oxidative stress could activate autophagy and induce apoptosis through a variety of signaling pathways [25,26]. However, a previous study indicated that short-term and low-concentration ROS treatment promoted autophagy, thus activating the cellular antioxidant response and protecting against oxidative injury, while long-term ROS exposure would result in irreversible autophagy flux injury and induce apoptosis [27]. Other studies also demonstrated that a high level of intracellular ROS induces the dysfunction of mitochondria and further damages lysosome function, thus impeding the formation of autophagy lysosomes and impairing autophagy flux [28]. After rapamycin-activated autophagy, ROS levels were significantly reduced, while 3-MA inhibited autophagy and increased the sensitivity of cells to H_2_O_2_ [29]. In this study, oxidative stress impaired the normal autophagy flux in hepatocytes, and the increasing lysosomal size also indicated a potential decrease in lysosomal function. Autophagy is a complex process involving dozens of proteins, like LC3II, Beclin1, and P62/SQSTM1. Beclin1 mediates the formation of autophagosomes, and LC3II binds to the autophagosome membrane; then, ubiquitin proteins are recruited through connected P62/SQSTM1, and finally, the autophagosome fuses with the lysosome for degradation [30]. The expression of Beclin1 and LC3II increases during the autophagy process, while the expression of P62/SQSTM1 initially increases but decreases with the degradation of the autophagosome [31]. The present study found that DHA reactivated H_2_O_2_-inhibited autophagy by promoting Beclin1 and LC3II expression, which was consistent with the degradation of P62/SQSTM1. To further prove these findings, 3-MA was used to block autophagosome formation, and the ROS-scavenging effect of DHA was reduced. This suggests that DHA-mediated antioxidant function is achieved by restoring the autophagy flux impaired by H_2_O_2_.

As the main organs of endogenous ROS production, the mitochondria are extremely vulnerable to ROS [32]. In liver oxidative stress, damaged mitochondria will further disrupt the redox homeostasis, leading to the production of more ROS, in a vicious cycle [33,34]. Our study found that H_2_O_2_ impaired mitochondrial membrane integrity, and increased the mitochondrial membrane permeability, which accompanied the loss of mitochondrial transmembrane potential (ΔΨm). After pretreatment with DHA, the function of mitochondria and the aberrant mitochondrial dynamics was rescued. These results suggest that DHA might reduce oxidative stress by alleviating mitochondrial damage. A study has shown that mitophagy plays an important role in repairing liver damage by inhibiting oxidative stress [35]. In the PINK/Parkin mitophagy pathway, PINK continues to degrade in a low expression state under normal conditions, while in the case of mitochondrial injury, PINK stabilizes on the mitochondrial outer membrane and recruits the E3 ligase Parkin to start mitophagy [36]. A previous study demonstrated that PINK/Parkin-mediated mitophagy protects against acute oxidative-injury-induced liver fibrosis [37]. Blocking the AMPK signaling pathway and thus repressing the PINK/Parkin-involved mitophagy would cause hepatocyte apoptosis and exacerbate NAFLD [38]. In this study, we found that DHA increased the expression of PINK1 and Parkin, and thus mitophagy was activated. The results of an immunofluorescence exam showed an increasing co-localization between mitochondria and PINK or LC3, which further demonstrated the occurrence of mitophagy. This is the first report on DHA activation of mitophagy in hepatocytes, and suggests that the protective mechanism of DHA in oxidative damage in hepatocytes may improve mitochondrial quality by activating PINK/Parkin-regulated mitophagy.

As a G protein-coupled receptor that can be specifically activated by DHA, GPR120 participates extensively in metabolic regulation by mediating the AMPK or MAPK pathways. Many studies have indicated that AMPK and the MAPK/ERK signaling pathway play an important role in the regulation of autophagy [39,40]. In our study, there was no significant change in AMPK signaling during H_2_O_2_-induced oxidative stress. However, the level of ERK1/2 was significantly inhibited in oxidative stress, and DHA treatment increased the expression level of ERK1/2. In the prevailing view, ERK1/2 promotes autophagy by negatively regulating mTOR signaling, and autophagosome formation would be attenuated with the inhibition of ERK1/2 [41,42]. However, another study stated that H_2_O_2_ induced cardiomyocyte injury and autophagy through the ERK1/2-dependent pathway [43]. These conflicting results may be related to different cell lines or different ways of intervening. In addition, the ERK1/2 signaling pathway has also been shown to be involved in the regulation of mitophagy by increasing the stabilization of PINK in MMO [44,45]. In the present study, when ERK1/2 was blocked by U0126, the activation of autophagy and mitophagy by DHA were inhibited. This suggests that DHA-mediated autophagy and mitophagy were regulated by ERK1/2, which is consistent with previous studies.

## 4. Materials and Methods

### 4.1. Cell Culture

The ALM12 cell (mouse normal hepatocyte) line was purchased from the BioVector NTCC Typical Culture Preservation Center (SCSP-550, Beijing, China). The cells were cultured in a DMEM/F-12 (1:1) medium (WISENT) containing ITS Liquid Media Supplement (WISENT, 315-081-XL), 10% (*v*/*v*) fetal bovine serum (Gibco, Grand Island, NY, USA), 100 U/mL penicillin, and 100 μg/mL streptomycin (Sigma, Shanghai, China) for three days at 37 °C with 5% CO_2_. AML12 cells were pretreated with DHA (50 μM, 53171, Sigma, Shanghai, China) for 12 h, followed with H_2_O_2_ (400 μM) challenging for 2 h. The cells collected were used in a cell viability assay, along with immunofluorescence, Western blotting, and flow cytometry tests.

### 4.2. Animals

Eight-week-old male C57BL/6J mice were purchased from the experimental animal center of Yangzhou University. Animals were housed at the Laboratory Animal Center, Nanjing Agricultural University, at a standard temperature (22 ± 2 °C) and in appropriate humidity (40–60%) on a 12 h light/dark cycle. The mice had ad libitum access to regular chow and water. One week later, to allow for acclimatization, all mice were randomly divided into three groups (*n* = 10). The DHA-treated group was given intragastric 50 mg/kg DHA, prepared as an emulsion with 1% Arabic gum. Intragastric gavage was applied 1× during a period of 7 days. Two hours after the last gavage, the DHA and CCl_4_ groups were intraperitoneally injected with 10% CCl_4_ (*v*/*v*) in olive oil at 2 mL/kg body weight to construct an acute liver injury model. The control group was intraperitoneally injected with an equal amount of neat olive oil. After 24 h, we sacrificed the animals. Blood was collected from the abdominal aorta and centrifuged at 3000× rpm for 10 min at 4 °C for serum collection. Serum was stored at −20 °C for subsequent analysis. The liver tissue samples were snap-frozen in liquid nitrogen and stored at −80 °C for molecular biological detection or routinely processed and 4% formalin-fixed for a histomorphology examination. All experimental procedures in our study were approved by the Animal Ethics Committee of Nanjing Agricultural University, China. Surgical and sampling procedures also complied with the “Guidelines on the Ethical Treatment of Experimental Animals” (2006) No. 398 published by the Ministry of Science and Technology, China, and with the “Regulations Regarding the Management and Treatment of Experimental Animals” (2008) No. 45, published by the Jiangsu Provincial People’s Government.

### 4.3. Biochemical Analyses

The levels of alanine transaminase (ALT), aspartate aminotransferase (AST), and lactate dehydrogenase (LDH) in serum were detected with an automatic biochemical analyzer (Hitachi 7020; HITACHI, Tokyo, Japan) using commercial assay kits (Wako Pure Chemical Industries, Ltd., Osaka, Japan).

### 4.4. Detection of SOD and GSH Levels in Mouse Livers

Liver SOD and GSH levels were measured using respective commercial assay kits (Jiancheng, Nanjing, Jiangsu, China) following the manufacturer’s instructions.

### 4.5. H&E Staining

Fresh liver samples were fixed in a 4% paraformaldehyde solution (Solarbio, Beijing, China). Then, these tissues were stained with hematoxylin and eosin to observe the morphology of the liver under a light microscope (BZ500; Olympus Corporation, Tokyo, Japan).

### 4.6. Immunofluorescence

Paraffin-embedded tissue sections were blocked with BSA for 2 h at room temperature and incubated with rabbit anti-LC3B (1:100; NB100-2220, NOVUS, Littleton, CO, USA) or PINK (1:100, AF7755, Beyotime, Shanghai, China) primary antibodies overnight at 4 °C, and then incubated with rabbit fluorescent secondary antibodies (1:200; Invitrogen, Carlsbad, CA, USA) for another 1 h at 37 °C. Cells were stained with the nuclear dye DAPI (1:1000, Solarbio, Beijing, China) for 5 min at room temperature and observed with a confocal microscope (Zeiss LSM 710 META, Jena, Germany).

### 4.7. Cell Viability Assay

A Cell Counting Kit-8 (CCK8, APExBIO, Houston, TX, USA) was used to analyze cell viability. Cells were seeded in 96-well plates and exposed to the treatments as indicated. Then, 10 μL of CCK-8 solution were added to each well for 4 h at 37 °C in the dark. Subsequently, the samples were analyzed by reading the optical density at 450 nm using a microplate reader (Bio-Rad, Hercules, CA, USA).

### 4.8. Cell Apoptosis Detection

Cells were harvested after different treatments and washed with PBS. Then, the cells were resuspended in 500 µL of buffer and stained with 5 µL of Annexin V FITC and 5 µL of PI (Solarbio, Beijing, China) for 15 min in the dark. The stained cells were detected by flow cytometry (BD, Franklin Lakes, NY, USA).

### 4.9. Intracellular ROS Detection

Intracellular ROS was detected using a DCFH-DA fluorescent probe (C2938, Invitrogen, CA, USA) following the manufacturer’s protocol. In brief, AML12 cells were seeded into 12-well plates at a density of 1 × 10^6^ per well. After being treated with H_2_O_2_, cells were harvested and washed with PBS. Then, cells were incubated with DCFH-DA (10 μM) at 37 °C in the dark for 15 min. The fluorescence intensity was detected by flow cytometry (BD, Franklin Lakes, NY, USA).

### 4.10. Autophagy Flux Detection

The mCherry-GFP-LC3 plasmid was kept by the Key Laboratory of Animal Physiology and Biochemistry Nanjing Agricultural University. The mCherry was used to label and track LC3, and the weakening of GFP could indicate the fusion of a lysosome and autophagosome to form an autophagosome. In the present study, AML12 cells were seeded on coverslips in 12-well plates, grown to 60–80% confluence, and transfected with a mCherry-GFP-LC3 plasmid using Lipofectamine 3000 (Invitrogen). Twenty-four hours later, the corresponding treatments were performed and observed using a laser confocal microscope (Zeiss LSM 710 META).

### 4.11. Mitochondrial Membrane Potential (MMP, ΔΨm) Assay

The mitochondrial membrane potential (ΔΨm) was measured using a mitochondrial- specific dual fluorescence probe, JC-1 (Yeasen, Shanghai, China). JC-1 is fluorescent probe widely used for the detection of mitochondrial membrane potential (ΔΨm). In healthy cells with high ΔΨm, JC-1 forms J-aggregates in mitochondrial matrix and emits a green fluorescence, while in low ΔΨm, JC-1 remains in the monomeric form in cytoplasm and emits an orange-red fluorescence. We can detect the change of fluorescence by flow cytometry to reflect the change of ΔΨm. Cells were stained with JC-1 to a final concentration of 5 mg/mL. After incubation for 20 min, the cells were washed twice with PBS, and detected by flow cytometry at a 488 nm and 562 nm excitation wavelengths.

### 4.12. Lysosome Tracker

AML12 cells were seeded in 12-well culture plates and grown to 60–80% confluence. The culture medium was removed and a LysoSensor™ Green DND-189 (Yeasen, Shanghai, China) probe was added in working concentration (1 μM). Cells were stained for 1 h. Staining medium was replaced with fresh normal medium, and then monitored by a laser confocal microscope (Zeiss LSM 710 META).

### 4.13. Western Blot Analysis

Tissue and AML12 cells were lysed with a RIPA lysis buffer (Beyotime) containing a protease inhibitor cocktail and a phosphate inhibitor cocktail (Sigma). After ultrasonic crushing, the sample was centrifuged for 30 min at 12,000× *g*, 4 °C. The supernatant was collected and the protein concentrations were determined using a BCA Protein Assay kit (Thermo Fisher, Rockford, IL, USA). Nuclear proteins were isolated using the specifications of a Nuclear and Cytoplasmic Protein Extraction Kit (P0028, Beyotime). Mitochondrial proteins were isolated using a mitochondrial protein extraction kit (G008, Jiancheng, Nanjing, China) according to the instructions. Whole protein samples were prepared by 5× SDS loading buffer, and then boiled for 10 min. Western blotting assays were performed as described previously. After being incubated with 4% (*w*/*v*) non-fat milk powder for 2 h at room temperature, the anti-LC3 (1:1000, AF5525, Beyotime), anti-Beclin1 (1:1000, AP0769, Bioworld), anti-P62/SQSTM1 (1:1000, 39749S, CST), anti-SOD1(1:1000, 37385S, CST), anti-HO-1(1:1000, sc-390991, Santa crus), anti-PINK (1:1000, AF7755, Beyotime), anti-Parkin (1:1000, AF7680, Beyotime), anti-PGC1α (1:1000, sc-518025, Santa crus), anti-Drp1 (1:1000, sc-101270, Santa crus), anti-AMPK (1:1000, BS6271, Bioworld), anti-P-AMPK (1:1000, 50081S, CST), anti-ERK1/2 (1:1000, 4370S, CST), anti-P-ERK1/2 (1:1000, 4695S, CST), anti-GAPDH (1:10,000, AP0063, Bioworld), and anti-β-Actin (1:10,000, AP0060, Bioworld) antibodies were used as primary antibodies incubated at 4 °C overnight. They were then washed by TBST, followed by incubating the blot with a secondary anti-mouse or anti-rabbit antibody for 2 h at room temperature. Bands were visualized by Super ECL Plus (Tanon, Shanghai, China) and analyzed by Image J software. Every experiment was repeated at least three times.

### 4.14. Statistical Analysis

All the experiments were duplicate and performed for three independent experiments. The data in our experiment are presented as mean ± S.EM. SPSS v. 16.0 software ((SPSS for Windows Release 16.0, SPSS Inc, Chicago, IL, USA) was performed for the statistical analysis. One-way ANOVA was used to analyze the significance differences between each group. A value of *p* < 0.05 was considered statistically significant.

## 5. Conclusions

In conclusion, our findings first demonstrate that DHA can induce protective mitophagy in oxidative-stress-induced hepatocyte damage and maintain mitochondrial function though the GPR120/ERK1/2 signaling pathway. Through these results, we proved the protective effect of DHA in acute liver injury, and revealed a new mechanism by which DHA alleviates oxidative stress in the liver.

## Data Availability

Data is contained within the article.
